# Insomnia as a concerned mental health issue during COVID-19 pandemic: A google trend analysis

**DOI:** 10.1192/j.eurpsy.2022.911

**Published:** 2022-09-01

**Authors:** D. Aniwattanapong, V. Phetsayanavin

**Affiliations:** 1 King’s College London, Institute Of Psychiatry, Psychology And Neuroscience, London, Thailand; 2 Chulalongkorn University, Department Of Psychiatry, Bangkok, Thailand; 3 Nakhon Phanom Ratchanakarin Psychiatric Hospital, Department Of Psychiatry, Nakhon Phanom, Thailand

**Keywords:** Insomnia, sleep, Covid-19, Google trend

## Abstract

**Introduction:**

Insomnia is one of the most common major health issues during the COVID-19 pandemic. There has been limited evidence that showed the correlation between insomnia and COVID-19 using Google trend.

**Objectives:**

To investigate the impact of the COVID-19 pandemic on interest in insomnia, including national mental health by a Google trend analysis that implicitly represents the state of distress and concern for this pandemic.

**Methods:**

We examined the Google trend search query data from these sleep-related keywords: insomnia, restless leg, and obstructive sleep apnea (OSA) from 1 Jan 2020 to 30 May 2020 and explored the correlation between the internet search volumes for insomnia and the cumulative number of new COVID-19 cases. In addition, we investigated the internet search pattern over time, before and during the COVID-19 pandemic.

**Results:**

During the early phase of the COVID-19 pandemic between January and May 2020, the Relative Search Volumes (RSV) curves showed that the cumulative number of new COVID-19 cases was significantly correlated with the rising search for these keywords linking to sleep-related conditions as follows: ‘insomnia’ (r = 0.41, p < 0.001), and ‘restless leg’ (r = 0.19, p = 0.009). However, it was not correlated with the keyword ‘OSA’ (r = -0.14, p = 0.07).

**Conclusions:**

These findings emphasize the impact of the COVID-19 pandemic on insomnia and the crucial need for public mental health interventions to be offered and accessible. The Google trend could be used as a new tool for public mental health surveillance in a new normal lifestyle.

**Disclosure:**

No significant relationships.

